# Exploring the Characteristics of Gut Microbiota Associated with Depression via the Depression Assessment Scales

**DOI:** 10.4014/jmb.2408.08042

**Published:** 2024-11-27

**Authors:** Seok Won, Eun-Ju Kim, Seong-Eun Park, Mee-Hyun Lee, Juhan Pak, Kyeongok Kim, Hong-Seok Son, Jae-Hong Kim, Suryang Kwak

**Affiliations:** 1 Department of Bio and Fermentation Convergence Technology, College of Science and Technology, Kookmin University, Seoul 02707, Republic of Korea; 2 Department of Biotechnology, College of Life Sciences and Biotechnology, Korea University, Seoul 02841, Republic of Korea; 3 College of Korean Medicine, Dongshin University, Naju 58245, Republic of Korea; 4 Department of Neuropsychiatry, College of Korean Medicine, Dongshin University, Naju 58245, Republic of Korea; 5 Department of Acupuncture and Moxibustion Medicine, College of Korean Medicine, Dongshin University, Naju 58245, Republic of Korea

**Keywords:** Depression, gut microbiota, assessment scales

## Abstract

Depression is a prevalent mental disorder with an increasing economic burden, and its pathogenesis remains poorly understood. Given the emerging evidence linking the gut microbiota to mental health, a better understanding of microbial profiles associated with depression is necessary. Here, we explore the association between gut microbiota and depression by utilizing 16S rRNA amplicon sequencing and depression assessment scales, including the Hamilton Depression Rating Scale (HDRS) and the Beck Depression Inventory (BDI). The study cohort consisted of 46 subjects, who were categorized into depression and normal groups based on medical diagnoses and depression scale scores. Our analyses revealed that HDRS-based classification better identified distinct gut microbiota structures associated with depression than medical diagnoses alone. Notably, lower beta diversity was observed in individuals with depression, indicating a more homogeneous gut microbial community. By employing both HDRS and BDI scores simultaneously, we identified specific taxa, such as *Bilophila* and *Alistipes*, which are linked to depressive symptoms. These findings highlight the potential of using depression assessment scales in conjunction with gut microbiota data to advance our understanding of depression and inform future treatment strategies.

## Introduction

Depression is one of the most common mental disorders worldwide, characterized by persistent feelings of sadness, hopelessness, and a lack of interest or pleasure in daily activities. A wide range of internal and environmental influences contribute to the occurrence of depressive symptoms, which affect millions of individuals every year [1, 2]. Although the average lifetime prevalence of depression ranges from 11 to 15% [3], it has significantly increased up to triple due to factors such as regional disparities and health crises, including the COVID-19 pandemic [4, 5]. Direct and indirect costs from the economic and social burden of depression are substantial among adults in the United States, with an estimated cost of $236 billion in 2018—a rise of more than 35% compared to 2010 [6]. Despite extensive research, the underlying mechanisms of depression remain unclear, and current treatment strategies are often insufficient [7]. Most antidepressants demonstrate only marginally better efficacy than placebo, leading to suboptimal responses in many patients using conventional treatments [7-9].

Measuring depression typically involves the use of standardized assessment tools and diagnostic criteria, including self-report questionnaires, such as the Hamilton Depression Rating Scale (HDRS), Beck Depression Inventory (BDI), EQ-5D-5L, and Visual Analogue Scale (VAS). HDRS is one of the most widely used scales for evaluating depressive symptoms and monitoring corresponding treatment progress in clinical settings [10-12]. It is considered a gold standard in clinical research and practice due to its comprehensive coverage of depressive symptomatology, including mood, guilt, suicidal ideation, insomnia, agitation, anxiety, weight loss, and somatic symptoms, along with its validated psychometric properties [13, 14]. BDI is another widely used and highly reliable measure for assessing the severity of depression. BDI consists of 21 multiple-choice questions that evaluate symptoms of depression, such as sadness, pessimism, past failure, loss of pleasure, and changes in sleep patterns [15, 16]. HDRS and BDI have been extensively validated in both clinical and research settings, making them trusted tools among medical professionals [13, 15]. The EQ-5D-5L is a self-reported health-related quality of life measure that assesses five dimensions, including depression [17]. In individuals with anxiety and depression, the EQ-5D-5L has demonstrated its reliable performance in measuring health-related quality of life, making it useful for both clinical and research settings [18-20]. The VAS has also been recently recognized as another useful tool for evaluating the severity of depression, particularly valued for its simple and fast administration [21, 22].

Recent studies have begun to explore the complex interactions between the gut microbiota and the brain, suggesting that the gut-brain axis plays a critical role in brain function and mental health [23-25]. The human gut carries a vast and diverse community of microorganisms, collectively known as the gut microbiota, which has been shown to influence various aspects of host physiology, including immune function, metabolism, and even behavior [26, 27]. Different patterns of gut microbiota have been observed across a range of psychiatric diseases, including depression, bipolar disorder, and schizophrenia [28, 29]. Emerging evidence indicates that alterations in the taxonomic composition and functionality of the gut microbiota may be associated with the development and progression of depression [30-32]. For instance, studies have identified differences in the abundance of specific microbial taxa between individuals with depression and healthy controls, suggesting that the alteration of gut microbiota architecture may contribute to the pathophysiology of depression, although the findings across the studies are not entirely consistent [33-38]. Given the potential role of the gut microbiota in modulating mood and behavior, understanding the specific microbial profiles associated with depression could provide valuable insights into novel therapeutic targets for depression. However, further research is still necessary to fully comprehend the underlying mechanisms and their therapeutic implications.

Thus, this study aims to investigate the association between gut microbiota and depression by assessing structural characteristics and specific taxonomic features in individuals diagnosed with depression compared to controls. The association among characteristics of gut microbiota, depression severity, and metadata were assessed from the study cohort consisting of a total of 46 subjects via 16S rRNA amplicon sequencing. We compared the medical diagnosis and depression assessment scales and demonstrated their potential for the association study of gut microbiota and depression. Finally, our analyses revealed more converged structures of gut microbiota within the depression group than the normal group and identified taxonomic features that distinguish individuals with depression from those without. These insights could contribute to the development of effective, microbiota-targeted interventions for mental health disorders.

## Materials and Methods

### Study Cohorts and Sample Collection

The medical ethics committee of Dongshin University Gwangju Korean Medicine Hospital approved the study protocol (DSGOH-2022-005). Individuals who had been experiencing symptoms of depression for at least two weeks prior to the recruitment period (from March to July 2023) were recruited as the cohort of this study at Dongshin University Korean Medicine Hospital. Those who were undergoing depression treatment or had a BDI score of 14 or higher were initially classified as depression patients. The additional HRDS and the EQ-5D-5L quality of life scale were also conducted subsequently. Then, the final determination of depression was made based on evaluations conducted following an interview with medical professionals. Fecal samples were collected at home within two weeks after the screening by patients and immediately shipped in a dedicated sterile package system to Dongshin University.

The initial cohort consisted of 50 subjects, each assigned an anonymous ID (R01 to R50). However, four participants (R12, R15, R33, and R43) were excluded from subsequent analyses due to withdrawal of consent or issues with fecal sample collection and sequencing.

### DNA Extraction and Sequencing

Metagenomic DNA was extracted from the pellet obtained by centrifuging 1 ml of the collected fecal samples at 13,000 rpm, 4°C for 10 min, using an AccuFAST automation system (AccuGene, Inc., Republic of Korea) following the manufacturer’s instructions. For the amplicon sequencing, the V4 region of the 16S rRNA gene was amplified from 1.5 ng of the extracted fecal DNA via 25 cycles of polymerase chain reactions using KAPA HiFi HotStart ReadyMix (Roche sequencing, USA) in triplicate with negative control (no template DNA) using barcoded 16S rRNA gene primers (515fb/806rb) containing Nextera adapters. The amplicons were purified with HiAccuBead (AccuGene Inc.) following the manufacturer’s instruction. The prepped amplicons were quantified using Miseq Reagent Kit v2 (Illumina, USA) for 500 cycles, pooled correspondingly, and sequenced via the Miseq sequencing platform (Illumina) with 2 × 250 bp paired-end reads.

### Data Processing and Analyses

The raw paired-end read data were trimmed and merged by the DADA2 [39], and taxonomic features were assigned from the domain to species levels using the Silva version 138.1 reference database [40] in QIIME 2 [41]. Sufficient sequencing depth for the taxonomic characterization of gut microbiota was secured via rarefaction analysis after the raw reads were incrementally rarefied in 10 levels within 0 to 25,000 reads. Diversities were assessed via the vegan [42] and ape [43] packages, and analysis results were visualized using ggplot2 [44] in R 4.1.0. Permutational multivariate analysis of variance (PERMANOVA) was conducted by adonis2 in the vegan [42] according to the Bray-Curtis dissimilarity and Jaccard index. Taxonomic features distinguishing normal and depression groups were identified using linear discriminant analysis effect size (LEfSe) [45], with an alpha value of 0.05 for the factorial Kruskal-Wallis test and a logarithmic LDA score threshold of 2.5.

## Results

### Collection of Study Cohorts and Their Data

The recruited cohort consisted of 25 normal subjects and 21 subjects with depression, which were classified by diagnostic results (Table 1). 16S rRNA amplicon sequencing was performed from the fecal metagenomes of the 46 subjects, and the resulting taxonomic data and metadata, including depression assessment scale scores, were analyzed. The depth of the demultiplexed sequences ranged from 45,688 to 81,414 reads (median 64,882 reads). Taxonomic feature observation frequency ranged from 32,980 to 65,570 (median 52,577) in all taxonomic ranks. The rarefaction analysis of both the Shannon index and richness confirmed the adequacy of sequencing read depths.

### The Structural Characteristics of Gut Microbiota Are Comparable between Subjects Diagnosed as Normal and Those Diagnosed with Depression

**Fig. 1:**
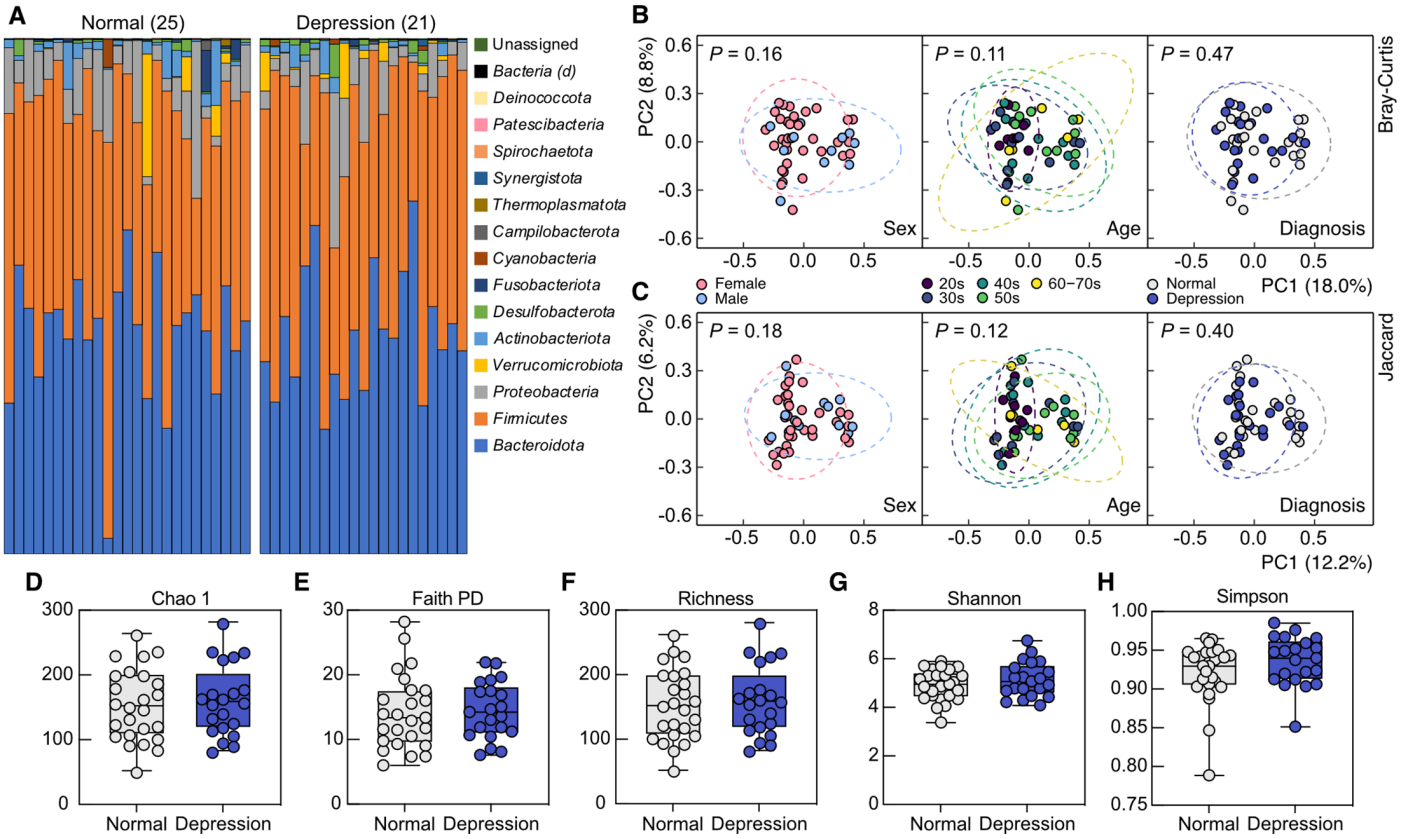
Characterization and comparison of the taxonomic structures of gut microbiota across subject groups. (**A**) Bar charts comparing taxonomic architectures of normal and depression subjects categorized by medical professionals. (**B–C**) Principal coordinate analysis about the structural dissimilarities of gut microbiomes of subjects grouped by their sex, age, and the medical diagnosis outcome, based on Bray-Curtis (**B**) and Jaccard (**C**) indices at the species level. *P* values of comparisons of the groups via permutational analysis of variance were presented in corresponding panels. (**D–H**) Alpha diversity comparisons between normal and depression groups with representative metrics. No statistically significant differences were found between the two groups across all metrics (Wilcoxon rank-sum test).

To elucidate depression-associated microbiota features of the gut microbiota, we first delved into the taxonomic data from the 16S rRNA sequencing together with the diagnosis results of depression by medical professionals and demographic metadata. The taxonomic composition of gut microbiota at the phylum level did not exhibit a visible distinction between normal and depression groups categorized by medical professionals (Fig. 1A). We further dissected the taxonomic data via principal coordinates analysis (PCoA) based on Bray-Curtis and Jaccard indices, and the structural dissimilarities among subject groups using PERMANOVA. Structural dissimilarities of the gut microbiota according to gender and age of the subjects were not statistically significant (Fig. 1B and 1C). This consistency of microbiota structures across demographics indicates less risk of demographic bias, which reduces the potential generalizability of the findings related to symptoms of depression. Still, the gut microbiota taxonomic structures between normal and depression groups were also not statistically distinct (Fig. 1B and 1C). In addition to the structural dissimilarities, we compared alpha diversity levels of individual gut microbiota between normal and depression groups, as it has been reported as a considerable factor associated with depressive symptoms [38, 46-48]. However, the two groups did not show significant differences in alpha diversity across various diversity metrics (Fig. 1D-1H). These results from gut microbiota structural analyses indicate that the normal and depressed individuals grouped via the medical diagnoses share comparable taxonomic characteristics of the gut microbiota.

### HDRS and BDI Scores Were Strongly associated with Medical Diagnostic Outcomes

**Fig. 2:**
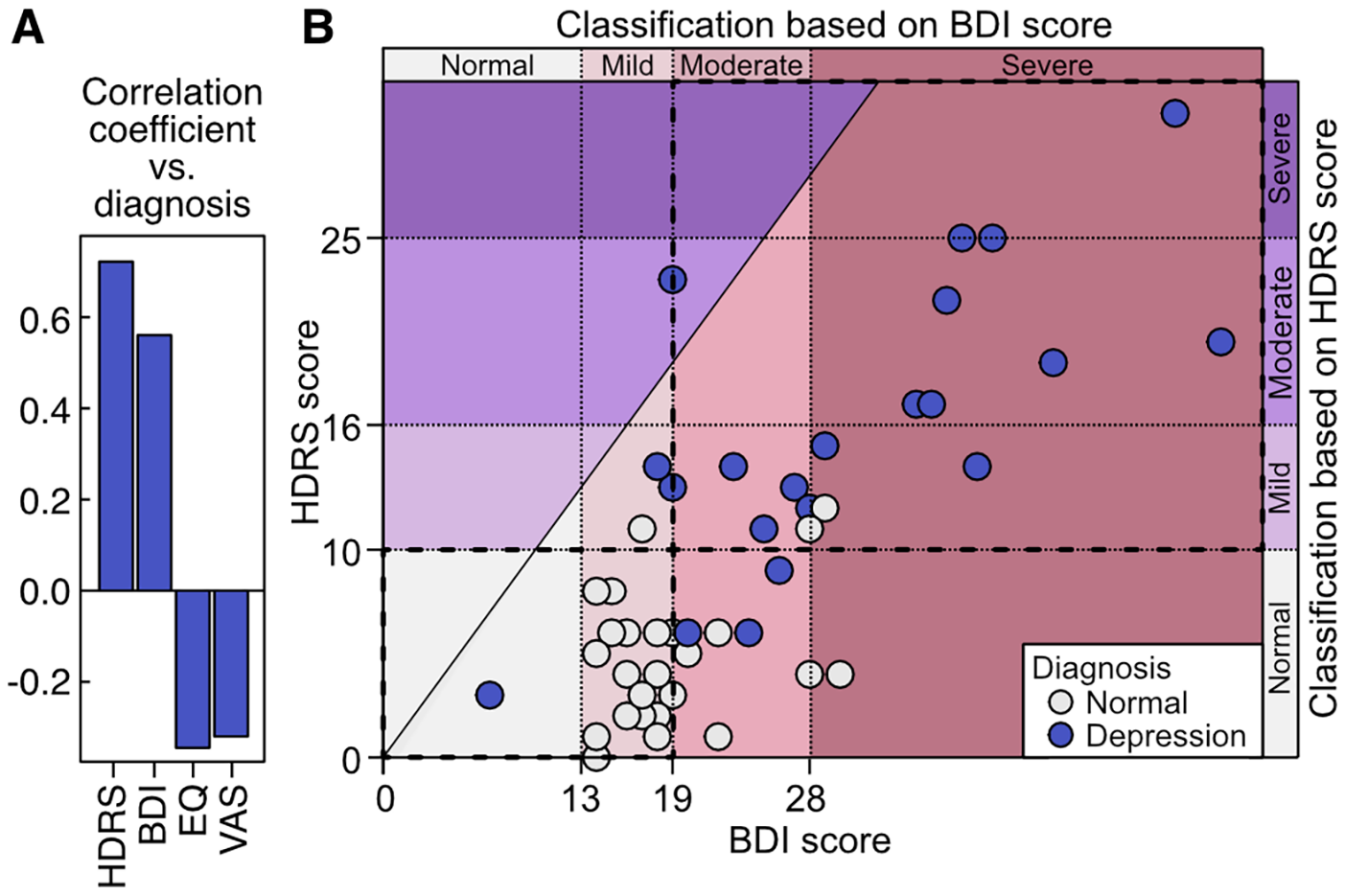
Correlation analyses among depression rating scales and the diagnosis by medical professionals. (**A**) Correlation coefficients (Point-Biserial Correlation) between the diagnosis grouping and each of four depression rating scales, namely Hamilton Depression Rating Scales (HDRS), Beck’s Depression Inventory (BDI), EQ-5D-5L (EQ), and Visual Analogue Scale (VAS). (**B**) Comparison of reclassified depression levels of subjects between the two selected depression rating scales, HDRS and BDI. The diagonal line in the plot indicates the points where the HDRS score and BDI score are equal (slope = 1).

The categorization based on the diagnostic results could not identify differences in the gut microbiota between the normal and depression groups. Thus, we took advantage of quantitative depression assessment scales, which are powerful tools in omics association studies [38, 49], along with medical diagnoses to define the association between microbial features and depressive symptoms using gut microbiota data. First, we assessed the correlations between medical diagnosis and depression assessment scales, namely HDRS, BDI, EQ-5D-5L, and VAS. As the outcome of the medical diagnosis is a binary variable, the correlation between the diagnostic results and depression rating scores via Point-Biserial Correlation. Scores derived from HDRS and BDI tests exhibited a strong correlation with the diagnosis results from medical professionals, while EQ-5D-5L and VAS outcomes showed weaker correlations compared to the two depression assessment scales mentioned above in the study cohort (Fig. 2A). HDRS showed the strongest association with medical diagnosis (coefficient = 0.72), whereas VAS showed the weakest (-0.32). We further investigated the results of HDRS and BDI tests for probing depression-associated gut microbiota characteristics, considering their noteworthy correlations with medical diagnosis. Although both HDRS and BDI scores showed an apparent association with the medical diagnosis, the assessment of depression severity by HDRS was more conservative than BDI (Fig. 2B). Concretely, among the total 46 subjects, HDRS identified 20 with depression (11 with mild, 8 with moderate, and 1 with severe depression). In contrast, BDI identified 45 subjects as having depression (21 with mild, 12 with moderate, and 12 with severe depression) and only 1 subject as normal.

### Subjects with Depression Classified by HDRS Exhibited Distinct Gut Microbiota Structures Compared to Normal Subjects

**Fig. 3:**
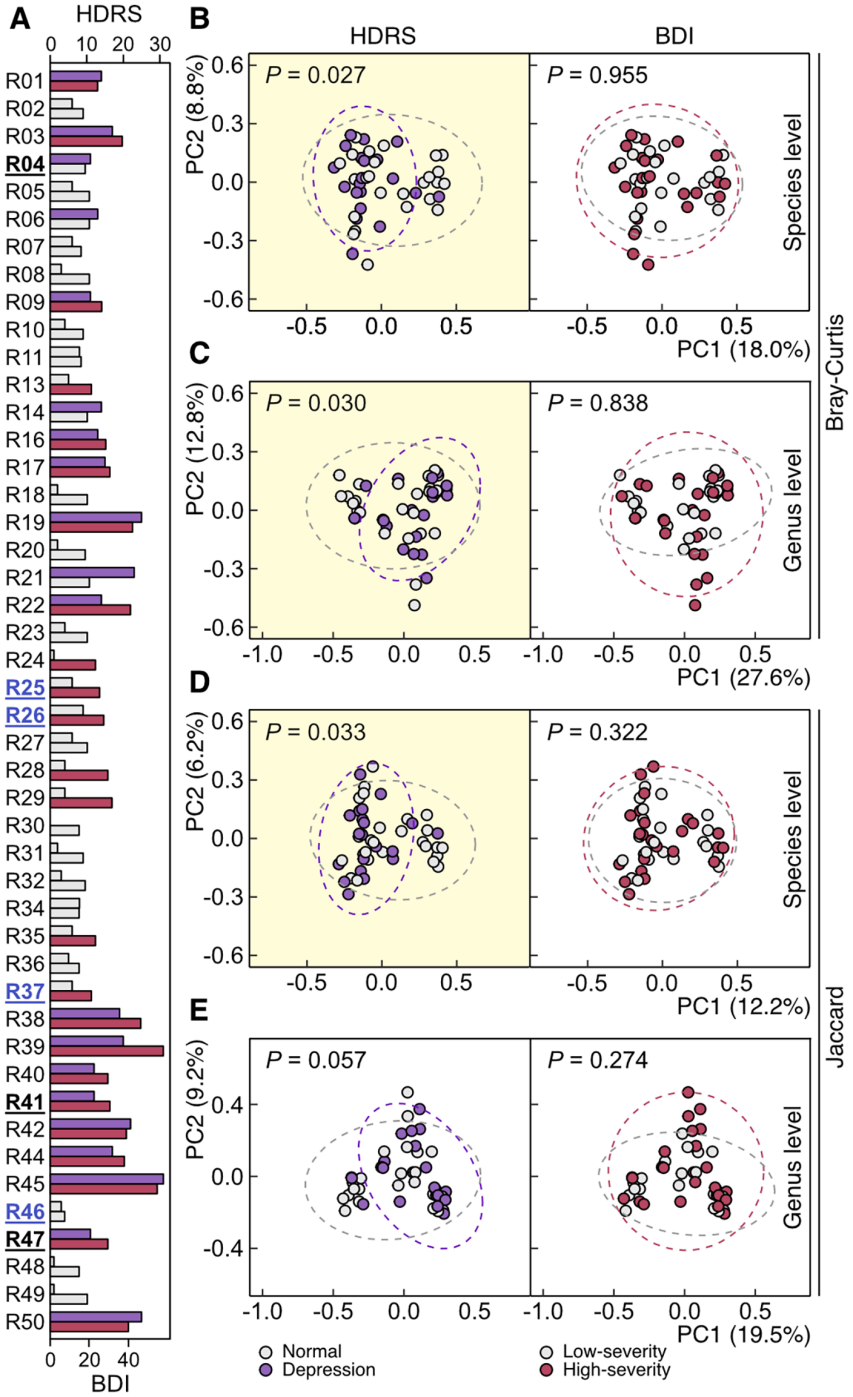
Structural differences in the gut microbiota between the two groups which were categorized by depression severity according to the Hamilton Depression Rating Scale (HDRS) and Beck Depression Inventory (BDI). (**A**) Categorization of each subject based on HDRS and BDI scores. The depression group categorized by HDRS (violet) includes subjects of all depression severity levels. Subjects whose classification based on HDRS conflicted with the medical diagnosis were underlined (black for subjects reclassified as depressed based on HDRS scores; blue for those reclassified as normal based on HDRS scores). In the case of categorization by BDI, the low severity group (gray) includes normal and mild depression subjects, and the high severity group (red) includes moderate and severe depression subjects. (**B–E**) Principal coordinate analysis of subjects’ gut microbiota taxonomic structures based on Bray-Curtis (**B** and **C**) and Jaccard (**D** and **E**) dissimilarities at both species and genus levels. The analyses exhibiting statistically significant differences between the two groups were highlighted in yellow (permutational multivariate analysis of variance, alpha value = 0.05).

We categorized the cohort into two groups per rating scale to determine the structural characteristics of the gut microbiota that were statistically correlated with the severity of depression assessed via HDRS and BDI. Based on the HDRS-based severity classification standard [50], the cohort was divided into normal (26 subjects) and depression (20 subjects of all depression severity levels) groups. In the BDI-based grouping, we categorized normal and mild depression subjects into the low-severity group (22 subjects), as only 1 subject was classified as normal according to the BDI score classification standard [15], and moderate and severe depression subjects into the high-severity group (24 subjects, Fig. 2B and 3A). Subjects with moderate and severe depression were categorized into the high-severity group (24 subjects, Fig. 3A). PCoA using Bray-Curtis dissimilarity described that taxonomic structures of gut microbiota at both species and genus levels were significantly distinct between HDRS-based normal and depression groups, while BDI-based low- and high-severity groups did not show structural dissimilitude (Fig. 3B and 3C). The same analyses with the Jaccard index led to similar patterns despite the lack of statistical significance on structural dissimilarity between HDRS-based normal and depression groups at the genus level (Fig. 3D and 3E).

Despite the significant correlation between medical diagnosis results and HDRS scores and corresponding similar categorization patterns (Fig. 2), only HRDS-based normal and depression categorization led to the successful distinction of gut microbiota taxonomic structures of depression subjects from those of normal subjects (Fig. 3B-3D), which medical diagnosis did not (Fig. 1B and 1C). The cohort reclassification based on HDRS scores was nearly identical to the medical diagnostic results except for 7 subjects whose reclassification brought about the statistically significant structural distinction of gut microbiota. Among the 7 subjects, 4 subjects (R25, R26, R37, and R46) were reclassified from depression to normal, and 3 subjects (R04, R41, and R47) were reclassified from normal to depression (Fig. 2B and 3A).

### The Gut Microbiota Was Less Diverse among Subjects with Depression Than among Normal Subjects

**Fig. 4:**
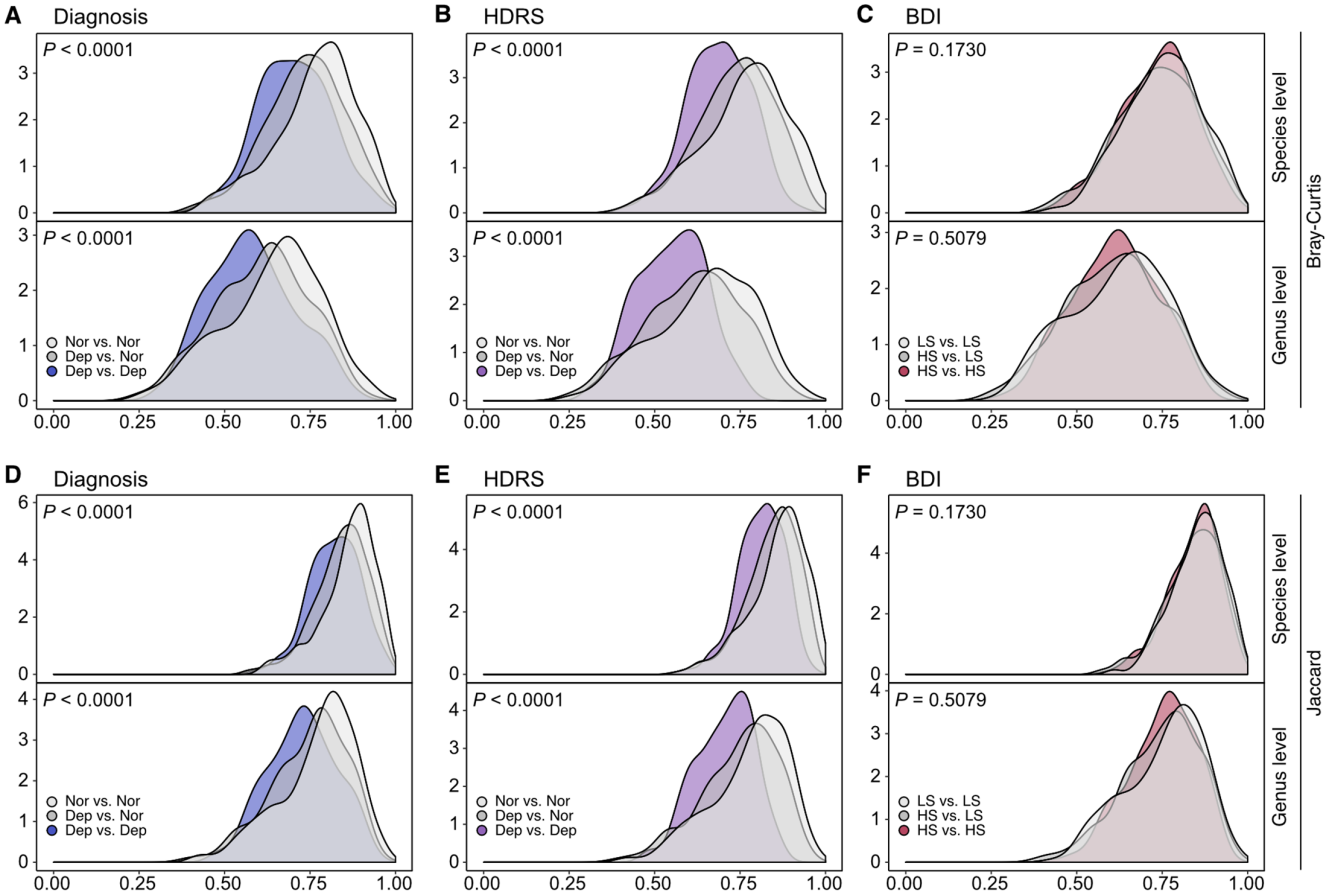
Inter-group and intra-group gut microbiota taxonomic dissimilarities based on the depression diagnosis, Hamilton Depression Rating Scale (HDRS), and Beck Depression Inventory (BDI). (**A–C**) Distribution of inter-group and intra-group Bray-Curtis dissimilarities. (**D–F**) Distribution of inter-group and intra-group Jaccard dissimilarities. (**A, B, D, E**) Nor, normal; Dep, depression; (**C, F**) LS, low-severity (normal and minor depression); HS, high-severity (moderate depression and severe depression). The three distributions of taxonomic dissimilarities were compared via the Kruskal-Wallis rank sum test.

To scrutinize the pattern of structural divergences associated with depression in detail, we investigated the group-level structural dissimilarities by reckoning and comparing taxonomic beta-diversities among subjects in a group and subjects between groups. In both diagnosis-based and HDRS-based categorization, intra-group Bray-Curtis dissimilarities among normal subjects were higher than those among subjects with depression, and inter-group dissimilarities (normal vs. depression) were located near the middle of the two intra-group dissimilarities (Fig. 4A and 4B). The same analyses with the Jaccard index led to similar patterns (Fig. 4D-4E). This consistency in beta diversity patterns indicates that the gut microbiota structures were less diverse among subjects with depression than among normal subjects. It means that there was less variation in the taxonomic structures between individuals within the depression group. The lower beta diversity in subjects with depression could be linked to the homogeneity of certain gut bacteria that are associated with depressive symptoms, although the current data set is inadequate to determine whether the more convergent gut microbiota structures resulted from depression or contributed to its development. Meanwhile, with the binary categorization of the cohort based on BDI scores, the distributions of the intra-group and inter-group dissimilarities were not distinct from each other, regardless of the beta-diversity indices used. It is consistent with the findings from PCoA, where the structures of subjects were not distinguishable after categorization based on BDI scores (Fig. 3). Results from both beta-diversity analyses with BDI-based categorization suggest that reclassifying into low- and high-severity groups based on BDI scores does not effectively identify depression-specific structural patterns in the gut microbiota within the current cohort.

### Identification of Taxonomic Features Specific to Subjects with Depression Reclassified by HDRS Scores

**Fig. 5:**
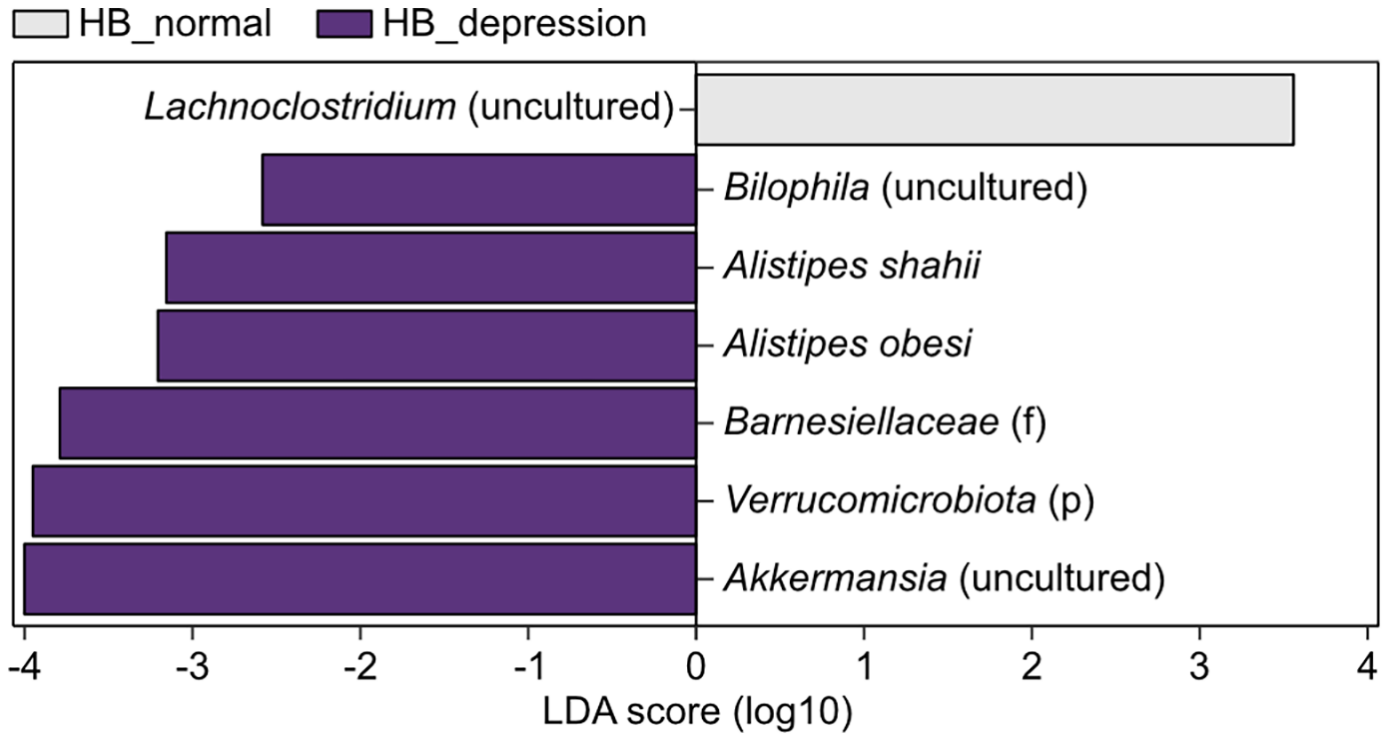
Identification of taxonomic features distinguishing HB-normal and HB-depression groups was determined from the gut microbiota of the selected subjects. Linear discriminant analysis effect size based on gut microbiota data of the selected subjects determined the specific taxonomic features at all taxonomic hierarchy levels. The HBdepression group exhibited four taxonomic features at the species level and one feature each at the family and phylum levels. No taxon was identified as a distinguishing feature in this analysis at the genus level.

To identify taxonomic features distinguishing subjects with depression from normal subjects, the gut microbiota was re-examined via LEfSe [45] including all taxonomic hierarchy levels. For more refined feature determination, we rigorously reclassified the cohort by simultaneously harnessing both depression assessment scales, namely HDRS and BDI. In particular, subjects who were included in both HDRS-based normal and BDI-based low-severity groups (Fig. 2B, lower left box) were reclassified as the HB-normal group. Those included in both HDRS-based depression and BDI-based high-severity groups (Fig. 2B, upper right box) were reclassified as the HB-depression group. Subjects simultaneously categorized in HDRS-based normal and BDI-based high-severity groups, or in HDRS-based depression and BDI-based low-severity groups, were excluded from this analysis. With the refined data, LEfSe identified a *Lachnoclostridium* sp. as a taxonomic feature distinctively abundant in subjects of the HB-normal group at the species level, although its specific species information was missing (Fig. 5). At the same time, *Bilophila* (undefined), *Alistipes shahii*, *Alistipes obesi*, and *Akkermansia* (undefined) were determined as gut microbiota features specific to subjects of the HB-depression group at the species level. Interestingly, *Bilophila* and *Alistipes* were previously reported as representative genera that are specifically abundant in the gut microbiota of patients with major depressive disorder [51], although our analysis could not identify any genus features, including the two genera, from the current data set. No studies have validated the association of the other screened taxonomic features with depressive symptoms to date.

## Discussion

In this study, we investigated the characteristics of the gut microbiota associated with depression by harnessing 16S rRNA amplicon sequencing of fecal samples and depression assessment scales—HDRS, BDI, EQ-5D-5L, and VAS—in addition to the medical diagnosis outcomes. Several prior studies have reported an inverse relationship between depressive symptoms and alpha diversity of gut microbiota [38, 46-48]. However, the current cohort did not show a significant difference in alpha diversity between individuals classified as normal or depressed based on medical diagnosis. Gau *et al*. similarly reported no statistically significant differences in alpha diversity indices between patients with depressive disorders and healthy controls in their meta-analysis and meta-regression of previous studies on gut microbiota composition [37]. Beta diversity of the cohort also did not show a statistically significant association with medical diagnosis outcomes in both Bray-Curtis and Jaccard indices. A gut microbiome-wide association study conducted by Radjabzadeh *et al*. involving two cohorts, each with over 1,000 participants, showed that the relationship between beta diversity and depressive symptoms varied depending on the cohort [38]. Although the consensus on the association between gut microbiota diversity and depression is limited, we found that the beta diversity levels among gut microbiota within the depression group were significantly lower compared to those of the normal group, as evidenced by the comparison of inter-group and intra-group beta diversity distributions. In other words, the subjects with depression share more converged gut microbiota structures. These findings also support the idea that depression is associated with specific alterations in the gut microbiota, which may play a role in the development or progression of depression, although individual variability and other factors such as diet, lifestyle, and medication use also play significant roles in shaping the gut microbiome [52, 53].

We hypothesized that the reclassification by the depression assessment scales performs better for the association study searching for depression-specific characteristics of gut microbiota as the scales provide a relatively more standardized and straightforward measure of depression severity directly from the subjects. Among the four scales, HDRS and BDI showed a considerable correlation with the medical diagnosis. On the other hand, although EQ-5D-5L and VAS have recently been appraised as valid depression-evaluating metrics [19-21], the correlations between their scores and medical diagnosis results were weaker than those of HDRS and BDI. This might be because HDRS and BDI were often referenced by medical experts in their depression diagnoses to assess the severity and presence of depressive symptoms [54, 55], including the current study. In the cohort of this study, the HDRS-based severity classification [50] led to more conservative depression severity categorization compared to BDI. HDRS-based normal and depression groups exhibited statistically distinctive gut microbiota architectures on PCoA, while medical diagnosis did not reveal such differences, corroborating that HDRS-based classification is an effectual tool for reclassification of depression for the purpose of gut microbiota association study. The HDRS classification probably led to a homogeneous grouping that aligns better with gut microbiota patterns via its direct and standardized measurement [14, 24, 50]. In contrast, medical diagnoses for depression could vary because medical professionals must consider multiple criteria, including scores from the depression assessment scales, for clinical judgment. These variations might lead to a heterogeneous grouping of individuals under the same medical diagnosis, where some may not display the typical gut microbiota profiles associated with depression [56], like the subjects who were reclassified from the depression group to the normal group based on their HDRS scores.

Our reclassification using both HDRS and BDI scores simultaneously identified specific taxonomic features uniquely associated with the depression group. The screened taxa included the genera *Bilophila* and *Alistipes*, which have been repeatedly recognized in previous studies as taxa linked to depression [34, 36, 51, 57]. Intriguingly, the association between depression and the abundance of the two genera has also been revealed indirectly via the Western diet. The Western diet is a risk factor for neuropsychiatric and psychological disorders, including depression [58]. It is characterized by high intakes of animal protein, as well as saturated fat and refined sugars, and these increase the abundance of *Bilophila* and *Alistipes* in the gut [58-60]. As both *Bilophila* and *Alistipes* are Gram-negative bacteria, their increased abundance might contribute to inflammation-related depressive symptoms through the activation of Toll-like receptor 4 by their lipopolysaccharides [61-63]. Also, the potential relationship between *Bilophila* abundance in the gut and mood-related disorders, validated in a rat model of spared nerve injury and its fecal microbiota transplantation into mice [64], can be another backend cause of the higher abundance of *Bilophila* in subjects reclassified in the HB-depression. Another possible mechanism is the disruption of the serotonin secretory system by higher abundance levels of *Alistipes*; it converts tryptophan into indole and, correspondingly, affects the availability of tryptophan, a precursor to serotonin biosynthesis [51, 65].

Despite multiple intriguing depression-associated gut microbiota features determined on the basis of depression rating scales, the relatively small cohort size was a considerable drawback limiting the depth of statistical assessments. Combining the current data with appropriate public depression-gut microbiome data, which should have been collected via an identical process with comparable depression rating scales, would validate the current findings substantially and expand the range of feasible analytical approaches, especially machine learning-based modeling. In addition to expanding the cohort size, incorporating additional variables affecting gut microbiota, such as diet, lifestyle, and medication history, would enable a more comprehensive and accurate understanding of the role of gut-brain axis in depressive symptoms.

In conclusion, the current study investigated the structural characteristics of the gut microbiota in relation to depression, utilizing both medical diagnoses and quantitative depression assessment scales. Reclassifying subjects based on HDRS scores—a rating scale strongly correlated with medical diagnoses—allowed us to observe distinct differences in gut microbiota structures between the depression and normal groups. In addition, lower beta diversity was noted among individuals with depression compared to normal individuals, in both cases of diagnostic result-based and HDRS-based categorization, suggesting a more homogeneous microbial community associated with depressive symptoms. Furthermore, by employing a refined classification method that combined HDRS and BDI scores, we identified specific gut microbiota features, such as certain species within the *Bilophila* and *Alistipes* genera, which have been previously linked to major depressive disorder. These findings underscore the potential of harnessing depression assessment scales in conjunction with robust clinical assessments and gut microbiota data to better understand the microbiota-depression relationship and its implications for future therapeutic strategies.

## Figures and Tables

**Table 1:** Descriptive statistics of the study cohort.

	Depression (*n* = 21)	Normal (*n* = 25)
Age: mean (±SD, range)	46.62 (±14.03, 24-72)	40.96 (±13.56, 22-67)
Sex (female%)	76.19	76.00
Smoking	1	3
Antidepressants (Yes)	7	1
Hamilton: mean (±SD, range)	15.67 (±7.02, 3-31)	4.84 (±3.26, 0-12)
BDI: mean (±SD, range)	30.52 (±11.85, 7-55)	18.96 (±4.9, 14-30)
EQ-5D-5L: mean (±SD, range)	0.76 (±0.15, 0.31-0.9)	0.84 (±0.08, 0.49-0.9)
VAS: mean (±SD, range)	60.71 (±18.12, 30-95)	72.00 (±16.07, 30-100)
